# Adiponectin-induced secretion of interleukin-6 (IL-6), monocyte chemotactic protein-1 (MCP-1, CCL2) and interleukin-8 (IL-8, CXCL8) is impaired in monocytes from patients with type I diabetes

**DOI:** 10.1186/1475-2840-5-17

**Published:** 2006-08-30

**Authors:** Sabine Abke, Markus Neumeier, Johanna Weigert, Gabriele Wehrwein, Elke Eggenhofer, Andreas Schäffler, Kevin Maier, Charalampos Aslanidis, Jürgen Schölmerich, Christa Buechler

**Affiliations:** 1 Department of Internal Medicine I, University of Regensburg, D-93042 Regensburg, Germany; 2 Institute of Clinical Chemistry and Laboratory Medicine, University of Regensburg, D-93042 Regensburg, Germany

## Abstract

**Background:**

Systemic adiponectin is reduced in patients with cardiovascular disease (CVD) and low adiponectin may contribute to the pathogenesis of atherosclerosis. However, circulating adiponectin is elevated in type 1 diabetes (T1D) patients, who have also a higher incidence to develop CVD. Because monocytes play an important role in atherosclerosis, we analysed the influence of adiponectin on cytokine and chemokine release in monocytes from T1D patients and controls.

**Methods:**

Systemic adiponectin was determined in the plasma and the high-molecular weight (HMW) form of adiponectin was analysed by immunoblot. Monocytes were isolated from T1D patients and controls and the adiponectin-stimulated release of interleukin-6 (IL-6), monocyte chemotactic protein-1 (MCP-1, CCL2) and interleukin-8 (IL-8, CXCL8) was analysed.

**Results:**

Systemic adiponectin was higher in T1D patients. Immunoblot analysis of the plasma indicate abundance of HMW adiponectin in T1D patients and controls. IL-6, CCL2 and CXCL8 secretion in response to adiponectin were found induced in monocytes from controls whereas only IL-6 was upregulated in T1D cells. The induction of IL-6 by adiponectin was abrogated by an inhibitor of the NFκB pathway.

**Conclusion:**

These data indicate that adiponectin-mediated induction of IL-6, CCL2 and CXCL8 is disturbed in monocytes from T1D patients and therefore elevated systemic adiponectin in T1D patients may be less protective when compared to controls.

## Background

The adipokine adiponectin (APM) is known to exert anti-inflammatory and insulin-sensitizing effects [1] but recent studies also describe proinflammatory activities of APM [2,3]. APM is highly abundant in human plasma and is secreted by adipose tissue in inverse relation to the body mass index [1]. APM circulates in blood as trimers, hexamers, and higher molecular weight (HMW) complexes [4]. A proteolytic cleavage product of adiponectin that includes its globular head group has also been detected in human plasma [5]. The biological activity of APM depends on its high order structure with different oligomeric complexes activating different signaling pathways. HMW-APM activates the NFκB pathway and consequently induces the secretion of IL-6 in differentiated THP-1 cells and primary monocytes [2,4]. Besides NFκB, HMW-APM also activates AMP-activated protein kinase (AMPK) [2] and stimulation of AMPK by metformin, a drug used in patients with impaired glucose tolerance, also induces IL-6 in cardiac fibroblasts [6]. Release of the chemokines CCL2 and CXCL8 is also stimulated by HMW-APM in human monocytes and may depend on NFκB activation [3,7].

Chemokines are molecules that attract cells of the immune system to the site of inflammation and also mediate the migration of monocytes to the subendothelium, an early event in the formation of atherosclerotic lesions. Like CCL2, CXCL8 is produced by a variety of cell types and is induced by proinflammatory mediators like endotoxin [8]. Both chemokines attract cells of the immune system to sites of inflammation and CXCL8 is unique among these proteins because of its high stability in-vivo [9]. The best characterized CC chemokine is CCL2 (MCP-1) and several studies suggest that CCL2 is the main chemokine involved in the recruitment of monocytes from blood into early atherosclerotic lesions. CXCL8 stimulates the adhesion of monocytes to endothelial cells and has also been linked to the development of atherosclerosis [10].

Whereas circulating adiponectin is reduced in the sera of patients with type 2 diabetes (T2D) and in patients with cardiovascular disease (CVD) [1], systemic adiponectin is elevated in type 1 diabetes mellitus (T1D), that is also associated with macro- and microvasculature complications [11,12]. Several studies demonstrate a disturbed response of T1D monocytes to endotoxin shown by an altered cytokine and chemokine secretion [13,14]. Because HMW-APM and endotoxin activate NFκB it might be suggested that T1D monocytes show an altered response to HMW-APM.

Monocytes are involved in the innate immune response and the formation of early atherosclerotic lesions. An altered cytokine and chemokine release from these cells may contribute to premature atherosclerosis and reduced immune function in T1D patients [15,16]. Therefore the influence of HMW-APM on the secretion of the multifunctional cytokine IL-6 and the chemokines CCL2 and CXCL8 was determined in monocytes of T1D patients and controls.

## Methods

### Patients and controls

Monocytes were purified from the blood of 10 female controls and 10 female T1D patients. The median age of the controls was 24 years (range 24 – 43) and of the patients 36.5 years (range 18 – 46). The mean body mass index (BMI) of controls was 20.6 kg/m^2 ^(range 17.5 – 22.3) and of the T1D patients 22.3 kg/m^2 ^(range 19.5 – 31). Only patients with a known history of T1D and an established therapy with intensive insulin treatment were recruited for the study. The median duration of diabetes was 13.5 years (range 7 – 34). The mean HbA1c was 7.2 % (range 5.7 – 9.5). Patients had no infectious disease within two weeks before blood was drawn. C-reactive protein was determined by an ELISA from Anogen (Ontario, Canada) in the plasma and was 1.94 mg/l (range 0.5 – 5) in controls and 1.1 mg/l (range 0.7 – 1.6) in T1D. All women gave informed consent and the study was approved by the local Medical Ethical Committee.

### Reagents

Macrophage SFM medium was from Gibco BRL (Karlsruhe, Germany). Recombinant M-CSF, recombinant human adiponectin, polyclonal adiponectin antibody and CXCL8 ELISA were from R&D Systems (Wiesbaden-Nordenstadt, Germany), IL-6 ELISA was from Pierce Biotechnology (Rockford, Illinois), and CCL2 ELISA was obtained from Amersham Biosciences (Freiburg, Germany). *E. coli *derived recombinant human proteins were used as standard for the IL-6, CXCL8 and CCL2 ELISAs. Apo E antibody was from Chemicon (Hampshire, U.K.). Vacutainer CPT were from Becton Dickinson (Franklin Lakes, NJ). InSolution™ NF-κB Activation Inhibitor was from Calbiochem (Darmstadt, Germany).

### Isolation and culture of primary blood monocytes

Peripheral blood leukocytes were isolated from 16 ml of whole blood by Vacutainer CPT and monocytes were further purified by magnetic separation with CD14 beads (Miltenyi Biotec, Bergisch Gladbach, Germany). Purity of the isolated monocytes was determined by flow cytometric analysis and was more than 98%. 500,000 monocytes were cultivated in 500 μl macrophage SFM medium in 24 well plates with 50 ng/ml M-CSF for 24 h. Subsequently the medium was replaced. Monocytes were either cultivated in 500 μl macrophage SFM medium with M-CSF or in the identical media supplemented with 10 μg/ml HMW-APM. Supernatants were collected 24 h later and used for ELISA.

### SDS-PAGE and immunoblotting

The plasma was diluted 1,000-fold in PBS and 10 μl were separated by SDS-polyacrylamide gel electrophoresis and were transferred to PVDF membranes (Bio-Rad, Germany). To analyse ApoE either 10 μg cellular lysate or 10 μl of the supernatants were used. Incubations with antibodies were performed in 1% BSA in PBS, 0.1% Tween overnight. Detection of the immune complexes was carried out with the ECL Western blot detection system (Amersham Pharmacia, Deisenhofen, Germany).

### Statistics

Data are represented as Box Plots indicating the median, the upper and lower quartile, the largest and the lowest value in the data set. Data are given as median values and the range of the values. Statistical differences were analyzed by two tailed Mann-Whitney U Test and a value of *P *< 0.05 was considered as statistically significant.

## Results

### Systemic adiponectin in the plasma of controls and T1D patients

**Figure 1:**
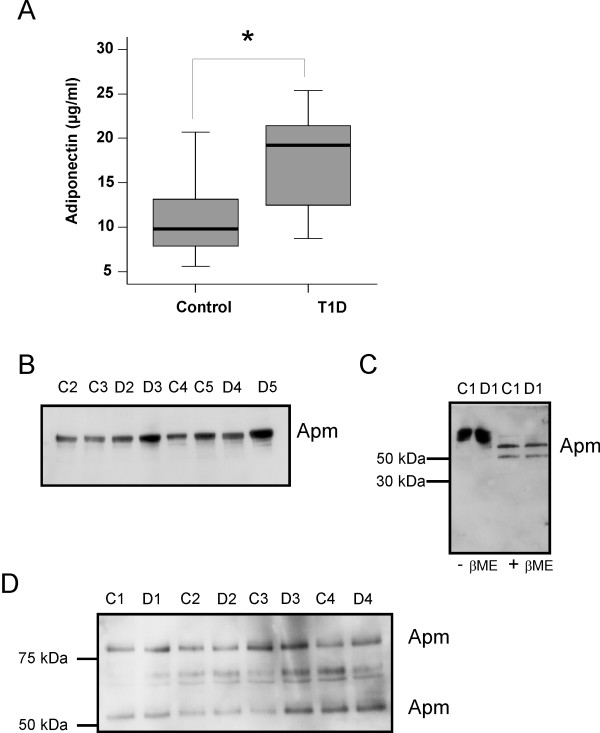
**Systemic adiponectin in T1D patients and controls**. (A) Systemic adiponectin was determined by ELISA in the plasma of 10 controls and 10 T1D patients. (B) Immunoblot of plasma adiponectin separated by SDS-PAGE under non-reducing conditions with plasma from controls (C2 to C5) and T1D patients (D2 to D5). (C) Immunoblot of plasma adiponectin separated by SDS-PAGE under non-denaturing (-βME) or denaturing conditions (+βME) from control 1 (C1) and T1D patient 1 (D1). (D) Immunoblot of plasma adiponectin separated by SDS-PAGE under reducing conditions with plasma from controls (C1 to C4) and T1D patients (D1 to D4).

Adiponectin was determined in the plasma of controls and was found to be higher in the patients. While the controls had 9.8 μg/ml (range 5.6–21.5), the T1D patients had 18.4 μg/ml (range 7.2–25.4) in their plasma (Figure 1A). The difference is significant with p = 0.04. Immunoblots were performed with plasma samples from controls and T1D patients which was seperated by SDS-PAGE under non-reducing conditions and HMW-APM was detected in all plasma samples (Figure 1B). Reducing conditions convert the HMW-APM to two protein subunits most likely resembling dimeric and trimeric APM (Figure 1C) and both multimers were detected in the plasma of the controls and T1D patients indicating a similar abundance of HMW adiponectin (Figure 1D).

### HMW-APM-induced IL-6 secretion in monocytes from controls and T1D patients

**Figure 2:**
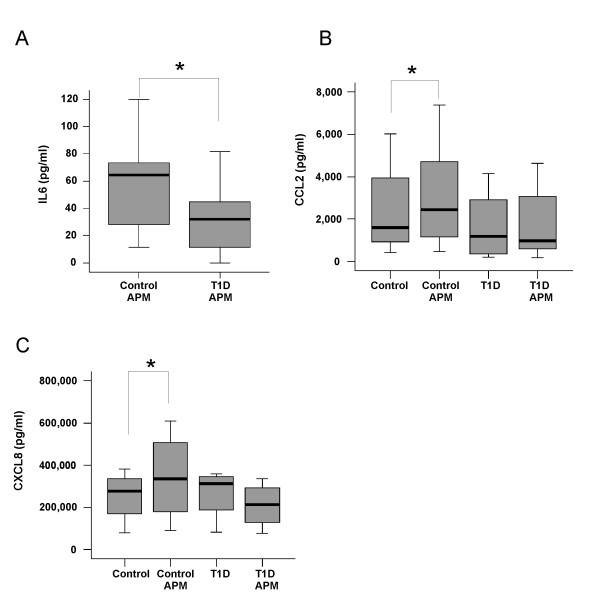
**HMW-APM stimulated IL-6, CCL2 and CXCL8 secretion of T1D and control monocytes**. Monocytes from 10 controls and 10 T1D patients were cultivated as described in the text. IL-6 (A), CCL2 (B) and CXCL8 (C) were determined in the supernatant of cells treated with 10 μg/ml HMW-APM for 24 h. CCL2 (B) and CXCL8 (C) were also determined in unstimulated monocytes.

IL-6 was determined in the supernatants of monocytes incubated with or without HMW-APM by ELISA. IL-6 was elevated in the supernatants of HMW-APM incubated cells. The median of HMW-APM induced secretion in monocytes from controls was 64.4 pg/ml (range 11.6–119.3) and in monocytes isolated from T1D patients 31.9 pg/ml (range 0–81.7) (Figure 2A). Therefore a significant lower IL-6 release in monocytes from T1D patients (p = 0.01) was observed upon HMW-APM treatment.

### HMW-APM-induced CCL2 secretion in monocytes from controls and T1D patients

The supernatants described above were also used to measure CCL2. CCL2 is constitutively secreted by non-stimulated monocytes and its concentration was 1,416 pg/ml (range 418–6,019) in controls and 1,198 pg/ml (range 208–3,227) in monocytes from T1D patients (Figure 2B) thus being similar in unstimulated monocytes isolated from controls or T1D patients. In HMW-APM-treated cells CCL2 secreted from control monocytes was 1,919 pg/ml (range 704–7,387) (p = 0.007 vs unstimulated cells) and from T1D 992 pg/ml (range 191–4,643) (p = 0.08 vs unstimulated cells) (Figure 2B).

### HMW-APM induced CXCL8 secretion in monocytes from controls and T1D patients

Furthermore CXCL8 was determined in the supernatants by ELISA. CXCL8 is already expressed in non-stimulated monocytes and its concentration was 276,000 pg/ml (range 78,500–382,000) in controls and 312,250 (82,500–358,000) in monocytes from T1D patients indicating a similar release of this chemokine (p = 0.47) (Figure 2C). CXCL8 in HMW-APM-activated monocytes from controls was 335,000 pg/ml (range 91,000 – 610,000) (p vs control = 0.02) and 213,500 pg/ml (range 77,000–334,500) in T1D monocytes (p = 0.13) (Figure 2C) and therefore was not induced by HMW-APM in monocytes from T1D patients.

### Metformin does not induce IL-6 in primary human monocytes

**Figure 3:**
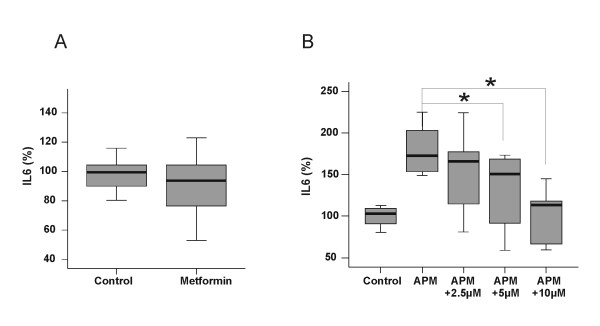
**Influence of metformin on IL-6 release and inhibiton of HMW-APM stimulated IL-6 by a NFκB inhibitor**. (A) Monocytes from 3 controls were cultivated with 0.5 mM metformin for 24 h and IL-6 was determined. (B) Monocytes from 2 different donors were incubated with 10 μg/ml HMW-APM alone or in combination with 2.5, 5.0 or 10.0 μM InSolution™ NF-κB Activation Inhibitor for 24 h and IL-6 was measured.

Monocytes from three different donors were incubated with 0.5 mM metformin for 24 h and IL-6 was determined in their supernatants. Our results demonstrate a similar release of IL-6 in unstimulated and metformin-treated monocytes (Figure 3A).

### Inhibition of NFκB abrogates HMW-APM stimulated IL6 release

Monocytes from 2 different donors were incubated with 10 μg/ml HMW-APM alone or in combination with 2.5, 5.0 or 10.0 μM InSolution™ NF-κB Activation Inhibitor for 24 h. IL-6 secretion in control treated cells was set to 100% and HMW-APM induced IL-6 release was 170% (range 149–225). Whereas 2.5 μM inhibitor did not significantly reduce IL-6 (166%, range 81–224), 5 μM reduce HMW-APM mediated IL-6 release to 144% (range 60–173, p = 0.02) and 10 μM reduce APM mediated IL-6 secretion to 116% (range 60–144, p = 0.0004) as is shown in Figure 3B.

### Apolipoprotein E is not altered by HMW-APM

**Figure 4:**
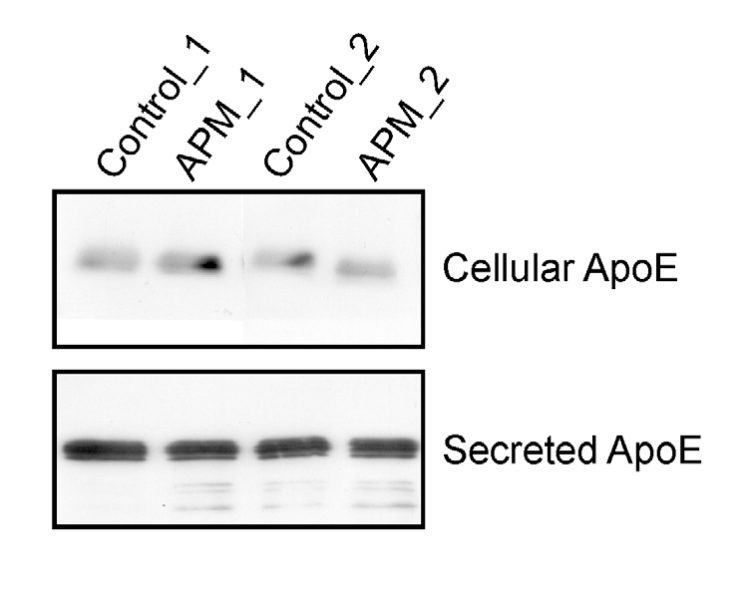
**HMW-APM does not regulate Apo E**. Cellular and secreted Apo E was analyzed by immunoblot in monocytes treated with PBS as solvent control or HMW-APM and the results for monocytes of 2 different donors is shown.

Apolipoprotein E (Apo E) secretion in monocytes is suppressed by endotoxin and therefore the influence of HMW-APM on intracellular Apo E was also studied. Neither intracellular nor secreted Apo E is altered by HMW-APM when monocytes from 3 different donors were analysed and the results from monocytes of 2 donors is shown (Figure 4) indicating that not all genes that respond to endotoxin are altered by HMW-APM.

## Discussion

Systemic adiponectin was found elevated in T1D patients [11,12] and this is also the case in the study group investigated in this work. HMW-APM is currently suggested to represent the biologic active form of adiponectin [17] and was detected in control and T1D plasma when analysed by immunoblot.

Several studies describe antiinflammatory effects of adiponectin in activated monocytes and *E. coli *produced full-length adiponectin at a concentration of 10 μg/ml was effective in reducing tumor necrosis factor α and IL-6 [18,19]. Full-length human adiponectin produced in a mouse cell line (10 μg/ml), however, induces IL-6, CXCL8 and CCL2 [2,7] in primary human monocytes whereas full-length human adiponectin produced in insect cells (1 μg/ml) has antiinflammatory properties [2]. In microvascular endothelial cells, full-length human adiponectin produced in the mouse cell line also upregulates CCL2 and CXCL8 whereas mesangial cells did not respond even when 50 μg/ml of adiponectin were used [7]. The recombinant globular adiponectin from mammalian cells (10 and 25 μg/ml were tested) did not upregulate CXCL8 and CCL2 in microvascular endothelial cells and furthermore could not block full-length adiponectin induced chemokine secretion [7]. A further study describes an induction of tumor necrosis factor α and IL-6 in macrophages by globular adiponectin (10 μg/ml) [20]. Adiponectin purified from human plasma stimulated CXCL8 in activated monocytes and inhibited CXCL8 in the presence of apoptotic cells [3].

The data from the literature indicate that the effects of adiponectin may depend on its high order structure, post-translational modification and on the activation state of the monocytes investigated. However, comparative studies have to be performed to identify the reasons for these different findings.

In the present study full-length human adiponectin produced in a mouse cell line was used and IL-6, CCL2 and CXCL8 were found induced in monocytes isolated from healthy controls.

When compared to monocytes isolated from controls, T1D cells show a reduced response to HMW-APM. Whereas HMW-APM stimulated secretion of IL-6 was lower in T1D monocytes, HMW-APM did not induce CCL2 or CXCL8 in T1D cells. Release of IL-6 and CCL2 is also lower in T1D cells when treated with endotoxin [14]. HMW-APM and endotoxin mediated increase of IL6 depends on the activation of NFκB indicating that NFκB signaling is partly impaired in T1D monocytes. In contrast, CXCL8 is similarily induced in T1D monocytes and control cells by endotoxin but not stimulated by HMW-APM in the patients' cells although upregulation of this chemokine, at least by endotoxin, also depends on NFκB [8]. Apo E is reduced by endotoxin [14] whereas HMW-APM did not alter Apo E in human monocytes. Therefore not all proteins are similarily regulated by endotoxin and HMW-APM.

IL-6 is higher in the sera of patients with T1D, T2D and in patients with coronary artery disease [21,22]. Therefore reduced IL-6 release of T1D monocytes upon stimulation with HMW-APM may be beneficial in the development of atherosclerosis. However, impaired IL-6 secretion may contribute to an ineffective innate immune response and higher incidence and duration of infections [23] and this may secondary promote atherogenesis.

In contrast to control monocytes, secretion of CCL2 is not altered by HMW-APM stimulation of T1D cells. Levels of CCL2 are increased early in the course of plaque formation and lead to increased monocyte migration into the atherosclerotic lesion [24]. Monocytes migrate into the intima due to a CCL2 concentration gradient that is formed by endothelial cells and monocytes [25]. Lower expression of CCL2 by T1D cells may increase this gradient and enhance migration of monocytes to the endothelium similar to a recent publication where an increase in CCL2 expression in adipose tissue enhances macrophage infiltration to this site [26]. Furthermore CCL2 is decreased in the plasma of T1D patients [27] and diminished secretion of CCL2 by monocytes may contribute to reduced plasma levels. The chemokine CXCL8 also activates monocytes and may recruit these cells to the endothelium. CXCL8 was found to be reduced by adiponectin purified from human plasma in macrophages when the cells were cultivated in the presence of apoptotic cells and was induced in the absence of apoptotic cells or when endotoxin was added to the macrophages. These authors suggest that adiponectin is a dual modulator of innate responses and not an antiinflammatory adipokine [3].

The current data indicate that HMW-APM signal transduction pathways are impaired in T1D monocytes. Therefore elevated circulating adiponectin may be less protective in T1D patients when compared to healthy individuals.

## Conclusion

Taken together, the present study demonstrates that T1D monocytes have a lower secretion of IL-6 and fail to induce CCL2 and CXCL8 when treated with HMW-APM. Therefore elevated systemic adiponectin in T1D patients may not reduce the risk to develop cardiovascular disease.

## Abbreviations

Apolipoprotein E (Apo E), high-molecular weight adiponectin (HMW-APM), interleukin-6 (IL-6), interleukin-8 (IL-8, CXCL8), macrophage colony stimulating factor (M-CSF), monocyte chemotactic protein-1 (CCL2, MCP-1), type 1 diabetes (T1D).

## Declaration of competing interests

The author(s) declare that they have no competing interests.

## Authors' contributions

SA and MN collected monocytes from controls and carried out immunoassays and immunoblots, JW carried out immunoassays, GW collected the patients and control samples and carried out immunoassays, EE carried out immunoassays, AS participated in the design of the study; KM carried out immunoassays, CA was involved in discussions and preparation of the manuscript and, JS is the head of the department and provided the required resources for research and was involved in fruitful discussions and preparation of the manuscript, CB conceived of the study, and participated in its design and coordination and prepared the manuscript.
